# Musculoskeletal manifestations of lower-extremity coccidioidomycosis: a case series

**DOI:** 10.5194/jbji-9-197-2024

**Published:** 2024-07-25

**Authors:** William Estes, L. Daniel Latt, Jacob Robishaw-Denton, Matthew L. Repp, Yash Suri, Tyson Chadaz, Christina Boulton, Talha Riaz

**Affiliations:** 1 College of Medicine, University of Arizona, Tucson, AZ, United States of America; 2 Department of Orthopedic Surgery, Banner – University Medical Center, Tucson, AZ, United States of America; 3 Department of Radiology, Banner – University Medical Center, Tucson, AZ, United States of America; 4 Valley Fever Center for Excellence, College of Medicine – Tucson, University of Arizona, Tucson, AZ, United States of America; 5 Department of Medicine, Division of Infectious Diseases, College of Medicine – Tucson, University of Arizona, Tucson, AZ, United States of America

## Abstract

**Background**: Coccidioidomycosis is a fungal infection endemic to the southwestern United States. Musculoskeletal manifestations are uncommon and seen in disseminated disease. While the involvement of the axial skeleton has been well described, the literature is limited on diseases involving the lower extremity. **Methods**: We identified three patients, at two regional academic medical centers in southern Arizona, who demonstrated different manifestations of osteoarticular coccidioidomycosis involving the lower extremity. **Results**: Case 1 is a 41-year-old male, with a history of HIV/AIDS and vertebral coccidioidomycosis, who presented with abscesses in the left hemipelvis and left proximal femoral osteomyelitis. He was treated with staged surgical debridement, including the use of amphotericin B impregnated beads. He remains on indefinite oral posaconazole suppression. Case 2 is a 46-year-old female, who presented with suspected right knee osteoarthritis. An MRI revealed septic arthritis and osteomyelitis. Necrotic bone was debrided, and synovial fluid cultures were positive for *Coccidioides*. She underwent a resection of the native knee joint with the insertion of an amphotericin B and voriconazole impregnated spacer. She continues oral itraconazole and awaits a total knee arthroplasty. Case 3 is a 76-year-old male, who presented with a draining right heel ulcer. Radiographs revealed bony destruction consistent with Charcot arthropathy. Irrigation and debridement revealed the gelatinous destruction of the talus and calcaneus, and cultures confirmed *Coccidioides* infection. A polymethyl methacrylate voriconazole spacer was placed. He subsequently underwent arthrodesis and remains on lifelong fluconazole. **Conclusion**: Lower-extremity osteoarticular coccidioidomycosis has various debilitating presentations that frequently mimic non-infectious etiologies. Treatment warrants surgical debridement, and prolonged antifungal therapy should be considered.

## 1 Introduction

Coccidioidomycosis is a fungal infection endemic to the southwestern United States and parts of Latin America. The causal organisms, *Coccidioides immitis* and *Coccidioides posadasii*, form spores from mycelia that live in the soil, and they become aerosolized when the soil is disturbed. If one or more spores are inhaled, many resulting infections cause no clinically significant illness. Of the one-third of infections that come to medical attention, most manifest as a pulmonary syndrome, often associated with rheumatic symptoms, rashes, and strikingly prominent fatigue (Ramadan et al., 2022). Up to 25 % of community-acquired pneumonia in endemic areas are attributable to coccidioidomycosis (Pu et al., 2023). Most of these illnesses last many weeks to several months but are eventually self-limiting (Ampel et al., 2009). A small proportion of infections, perhaps 0.5 %, progress by hematogenous spread beyond the thorax. Known as disseminated coccidioidomycosis, this complication most commonly produces one or more destructive lesions of the dermis, the meninges, or the musculoskeleton (Stockamp and Thompson, 2016). Although immunocompromised patients are at particular risk, most patients who develop disseminated coccidioidomycosis are not overtly immunocompromised, and their susceptibility to this complication may be due to more selective and complex immunogenetics (Stockamp and Thompson, 2016; Ampel, 2007; Rammaert et al., 2014; Reach et al., 2010; Galgiani et al., 2023).

Musculoskeletal manifestations of coccidioidomycosis include osteomyelitis, tenosynovitis, septic arthritis, and muscular abscess (Ramanathan et al., 2019; Weisenberg, 2018; Taxy and Kodros, 2005; Stockamp and Thompson, 2016; Rammaert et al., 2014; Nasrawi et al., 2020; Li et al., 2014; Ahmad et al., 2021). Multiple lesions are not uncommon, and the frequently affected areas are the knee and the spine (Ahmad et al., 2021; Kim et al., 2022; Naeem et al., 2022; Zeppa et al., 1996; Li et al., 2014; Weisenberg, 2018; Waterman et al., 2010; Taxy and Kodros, 2005; Reach et al., 2010; El Abd et al., 2012; Kakarla et al., 2011; Ramanathan et al., 2019; Rammaert et al., 2014; Moni et al., 2023). The foot and ankle, shoulder, and wrist are less common, and the involvement of the craniofacial bones and bone marrow has been described rarely (Fishco and Blocher, 2000; Naeem et al., 2022; Nasrawi et al., 2020; Narla and Narla, 2021; Antony et al., 2015). Musculoskeletal lesions may be accompanied by purulent discharge and sinus tracts that suggest infection, but frequently, they can be easily confused with non-infectious etiologies such as neoplasm, tendonitis, or osteoarthritis (Nasrawi et al., 2020; Fishco and Blocher, 2000; Li et al., 2014; Taxy and Kodros, 2005).

Given the paucity of published reports of osteoarticular coccidioidomycosis involving the lower extremity, in this case series, we selected three patients with destructive skeletal coccidioidomycosis involving their lower extremities and discuss the treatment and management strategies.

## 2 Methods

In a PubMed search (not a systematic review) of case reports and case series from 2010 to 2022 containing keywords of “musculoskeletal” and “coccidioides”, “coccidiomycosis”, or “coccidioidomycosis,” seven prior cases of lower-extremity coccidioidomycosis were identified (Table 1).

**Table 1:** Cases described in the literature on lower-extremity musculoskeletal coccidiomycosis. Lower-extremity musculoskeletal coccidiomycosis presentations and management based on case reports available on PubMed. Note that N/A stands for not applicable.

Case	Age/sex	Site	Risk factors	Suspected pathology	Coccidioidomycosis diagnosis	Surgical treatment	Antifungal regimen	Complications	Follow-up	PubMed ID
Case 1	41/M	Hip/thigh	HIV/AIDS, endemicarea	Abscess/septic arthritis	Wound cultures	Repeat irrigation and debridement with amphotericin beads	10 d of liposomal amphotericin 5 mg kg^-1^ followed by indefinitedaily triazole therapy	Osteonecrosis offemoral heads,recurrent abscesses	2 years	N/A
Case 2	46/F	Knee	Endemic area	Osteoarthritis	MRI followed by synovial cultures	Irrigation and debridement	Fluconazole 400 mgdaily, indefinitely	Extensive joint damage and collapse of lateral tibial plateau	2 years	N/A
Case 3	73/M	Foot/ankle	Diabetes mellitus type II,endemic area	Charcot arthropathy	Nucleic acid hybridization and culture of bone biopsy	Irrigation and debridement with voriconazole beads	Fluconazole indefinitely	Hindfoot fusion withmultiple revisions	5 years	N/A
Fishco andBlocher (2000)		Foot/ankle		Tendinitis	Bone culture					11107712
Li etal. (2014)	27/M; 78/M	Patella	Previous pulmonarycoccidioides infection,endemic area	Bone neoplasm	Surgical biopsy histology in both cases; bone culture in second case	Bone and soft tissuedebridement	2 weeks of fluconazole 800 mg, 1 year of fluconazole 400 mg, 6 months of oral fluconazole	None	6 weeks,1 month	24548622
Taxy andKodros (2005)	22/M	Foot/ankle	Endemic area	Posttraumatic arthritis	Synovial biopsy and culture	Arthrotomy anddebridement	14 months of fluconazole	Chronic pain	14 months	16203277
Ahmad etal. (2021)	49/M	Knee	Previous pulmonarycoccidioides infection,endemic area	Septic arthritis	Arthrocentesis cultures	Arthrotomy,debridement, anddrainage	2 d of liposomal amphotericin B, indefiniteitraconazole 400 mg	None	6 months	34141676
Nasrawi etal. (2020)	29/M	Knee	Suspected previous pulmonary coccidioidesinfection, endemic area	Inflammatory arthritis	Arthrocentesis histology and cultures	None	Daily IV amphotericin B for 14 d, then three times per week for 12 weeks	Lost to follow-up	12 weeks	33238746
Waterman et al. (2010)	11/M	Patella			Bone biopsy	Bone and soft tissue debridement	Long-term antifungals			20415313
Weisenberg (2018)	78/M	Knee	Suspected previous pulmonary coccidioidesinfection, endemic area	Osteoarthritis	MRI followed byarthrocentesis cultures	Multiple debridements, synovectomy, and partial meniscectomy	5 months of fluconazole800 mg, 3 years of itraconazole 200 mg, thenlifelong fluconazole200 mg	Joint stiffness	3 years	29535094

A case series was designed by including three patients at two academic medical centers in southern Arizona who demonstrated different manifestations of disseminated coccidioidomycosis in the lower extremity. Each of these patients was treated between 2018 and 2023 by orthopedic and infectious disease specialists. The patients' electronic medical records were reviewed to determine the initial presentations, diagnostic tests, different treatments, and disease courses.

## 3 Results

### 3.1 Case 1

A 41-year-old male, with a history of HIV/AIDS and prior pulmonary coccidioidomycosis complicated by a thoracic paraspinal abscesses status post-surgical debridement maintained on fluconazole at 400 mg d^-1^, presented with a sinus tract on his left lateral hip that drained a purulent discharge. He was non-adherent with his antiretroviral therapy (elvitegravir, cobicistat, emtricitabine, tenofovir, and darunavir). His CD4 (cluster of differentiation 4 T cells) count was 140 mm^-1^, and the viral load was 40 copies per milliliter.

**Figure 1:**
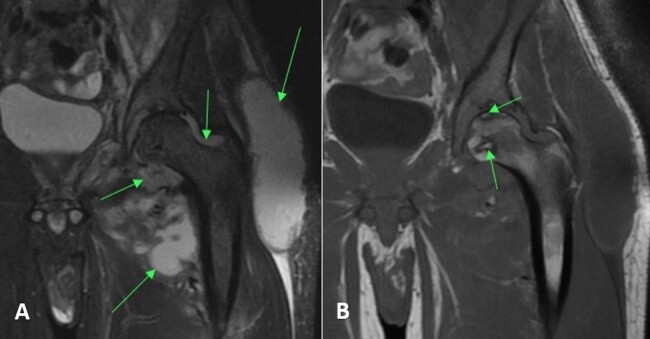
Case 1 hip MRI without contrast demonstrating **(a)** multiple abscesses and fluid collection (arrows) in the left hemipelvis with communicating septic arthritis of the hip on the T2-weighted (MRI sequence sensitive to tissue with high water content) sequence and **(b)** osteonecrosis of the femoral head (arrows) on the T1-weighted (MRI sequence sensitive to tissue high in lipid and protein) sequence.

An MRI of the left lower extremity with and without contrast demonstrated multiple abscesses surrounding the left proximal femur with extension into the paraspinal musculature, left acetabular erosion, and abscess within the left iliopsoas with gas (Fig. 1). His *Coccidioides* complement fixation (CF) titer was 1:256 .

**Figure 2:**
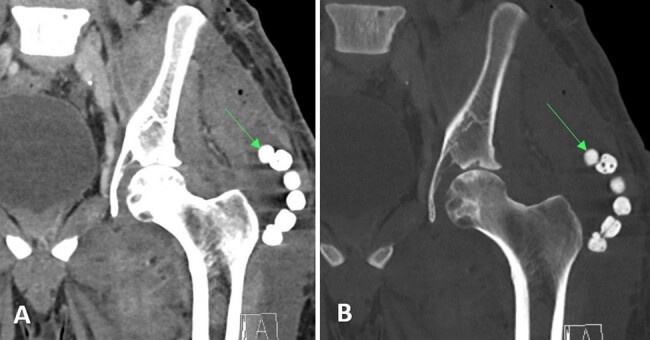
Case 1 post-op CT scan of the pelvis demonstrating antibiotic beads (arrows) in the left thigh.

He was started on intravenous liposomal amphotericin B (5 mg kg^-1^) for 10 d. He underwent a surgical debridement of the left thigh and flank abscesses and left hip arthrotomy and a washout. Fungal wound cultures confirmed *Coccidioides* infection. In total, 33 methyl methacrylate cement beads containing 1 g vancomycin, 1 g cefepime, and 150 mg amphotericin B deoxycholate were placed in the abscess cavities (Fig. 2). He was discharged on fluconazole at 800 mg as the patient was considered to have a breakthrough infection at 400 mg of fluconazole previously.

He presented to the ED (emergency department) again for left hip pain and fluctuance 9 months later. A repeat MRI with and without contrast of the left lower extremity demonstrated findings consistent with abscess and osteomyelitis of the same distribution as previously but with a significantly decreased size. The *Coccidioides* CF titer remained at 1:256 . The patient underwent further irrigation and debridement of his left posterior thigh. He was switched to posaconazole (300 mg daily) due to the clinical failure of fluconazole. At the 2-year follow-up, he was doing well and was without any recurrence of the abscess.

### 3.2 Case 2

**Figure 3:**
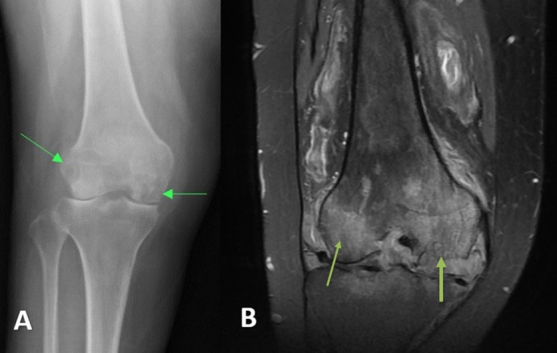
Case 2 **(a)** anterior–posterior right knee radiograph demonstrating subchondral lucency (green arrows), suggestive of advanced osteonecrosis and joint space narrowing consistent with arthritis. **(b)** T2-weighted sequence coronal section right knee MRI demonstrating cortical erosion suggestive of osteomyelitis (yellow arrows), with a mass-like synovial proliferation suggestive of atypical septic arthritis and full-thickness chondrolysis of the medial and lateral compartments.

**Figure 4:**
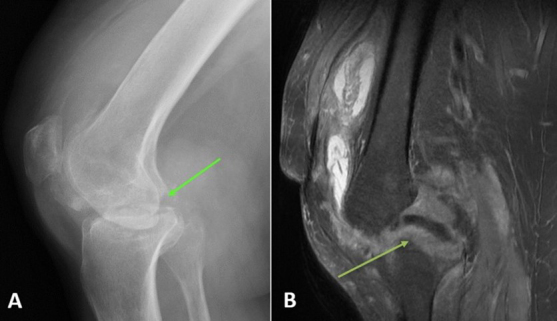
Case 2 status post-irrigation and debridement. **(a)** Lateral right knee radiograph demonstrating destructive changes in the medial femoral condyle (green arrow) and joint space narrowing. **(b)** T2-weighted sequence sagittal knee MRI demonstrating the collapse of the lateral tibial plateau (yellow arrow), increased synovial thickening, and further maceration of the menisci and cruciate ligaments.

**Figure 5:**
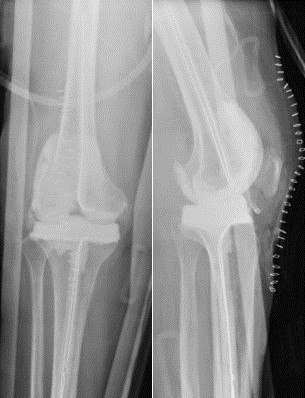
Case 2 anterior–posterior and lateral knee radiographs demonstrating post-surgical changes from the right total knee joint resection and antibiotic impregnated cement and intramedullary rods in the distal femur and proximal tibia.

A 46-year-old female with history of methamphetamine use, obesity (BMI >40 ), and a history of right tibial–fibular fracture presented to an orthopedic specialist with right knee pain and swelling for over 2 years. Radiographs demonstrated tricompartmental degenerative changes. This was followed by multiple corticosteroid injections with no relief. Then, 1.5 years later, a right lower-extremity MRI without contrast showed medial and lateral femoral condylar cortical erosions, extensive osteomyelitis with cartilage destruction, and femoral subperiosteal abscess (Fig. 3). Synovial fluid routine aerobic cultures came back positive for *Coccidioides* species. She underwent arthroscopic irrigation and debridement of the right knee and was found to have osteonecrosis of medial femoral condyle. A bone biopsy demonstrated necrotizing granulomatous inflammation with abundant spherules that is consistent with *Coccidioides* infection. She was discharged on oral itraconazole (400 mg d^-1^) and later switched to fluconazole at 400 mg d^-1^ due to insurance issues. At the 4-months follow-up, she reported increasing pain in her right knee. *Coccidioides* complement fixation titers were unchanged at 1:128 . The right knee MRI with and without contrast demonstrated the progression of osseous destruction involving tibial plateau (Fig. 4). She underwent a resection of the infected articular femoral and tibial bone and received a placement of an articulating antifungal cement spacer containing 4 g vancomycin, 400 mg amphotericin B deoxycholate, 800 mg voriconazole, and 4.8 g tobramycin (Fig. 5). She was discharged on fluconazole at 800 mg d^-1^, but 7 weeks later, she represented with purulent knee drainage and underwent a spacer exchange. She is currently on itraconazole (200 mg every 8 h), and the most recent CF titer is 1:32 .

### 3.3 Case 3

**Figure 6:**
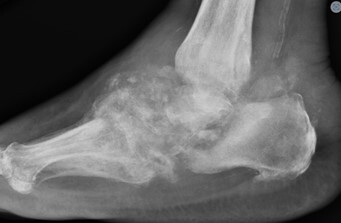
Case 3 lateral foot radiograph showing severe destructive arthropathy with signs of superimposed soft tissue infection and possible osteomyelitis.

**Figure 7:**
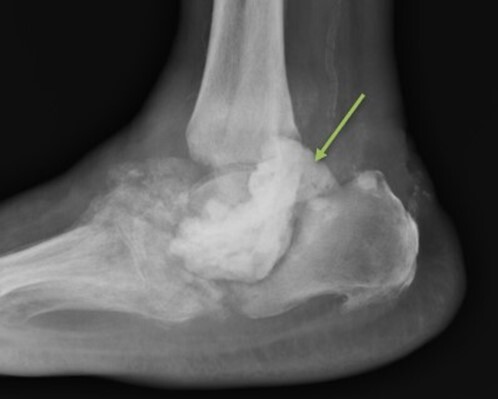
Case 3 sagittal foot radiograph demonstrating post-operative changes, including the placement of an antifungal cement spacer (arrow).

A 73-year-old man with insulin-dependent diabetes mellitus and peripheral neuropathy had 6 months of right lower-extremity swelling and erythema presumed to be caused by Charcot arthropathy. He went on to develop a right medial ankle ulcer with drainage. Right ankle radiographs demonstrated extensive destruction of the talus with varus deformity and signs of superimposed soft tissue infection (Fig. 6). He underwent irrigation and debridement, during which minced gelatinous bone and soft tissue were found throughout the hindfoot with a complete destruction of the talus and partial loss of the calcaneus. Multiple specimens of bone and soft tissue grew *Coccidioides* species. A polymethyl methacrylate spacer containing vancomycin, tobramycin, and calcium sulfate beads with 200 mg voriconazole were used to fill the structural defect (Fig. 7) once a *Coccidioides* infection was confirmed. Further investigation into the patient's records revealed a history of pulmonary coccidioidomycosis 3 years prior thought to have been eradicated with a 6-month course of fluconazole. The *Coccidioides* CF titer was 1:4 . He was placed on oral fluconazole at 400 mg d^-1^.

**Figure 8:**
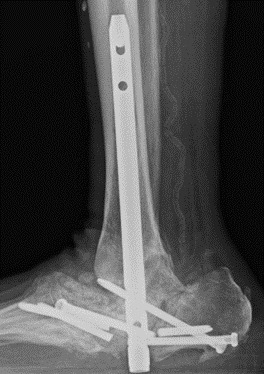
Case 3 lateral ankle radiograph showing nonunion and hardware failure after tibiotalar arthrodesis.

**Figure 9:**
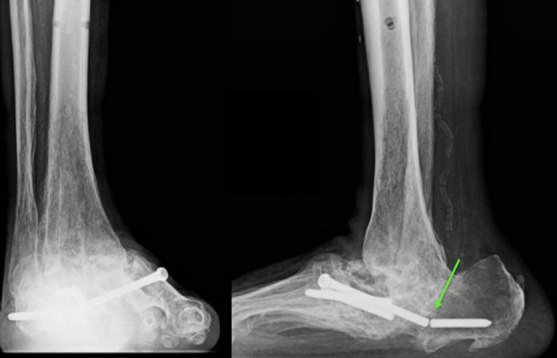
Case 3 mortis and lateral ankle radiographs showing tibiocalcaneal nonunion with a broken screw (arrow).

Then, 9 months later, the patient underwent the removal of the antibiotic spacer and tibiotalar calcaneal arthrodesis. Repeat fungal cultures were positive for *Coccidioides* species, and serial imaging showed bony nonunion. (Fig. 8). A revision hindfoot arthrodesis was performed 18 weeks later, during which intraoperative cultures grew *Coccidioides* species from the hindfoot nonunion site. He was continued on fluconazole at 400 mg d^-1^. Then, 4 years after the initial presentation, the patient developed an ulceration on the plantar surface of his foot that was caused by the erosion of the arthrodesis nail through the soft tissues of the heel (Fig. 9). He then underwent the surgical removal of the nail with the placement of calcium sulfate beads containing 200 mg voriconazole and 1 g vancomycin into the intramedullary canal. Fungal cultures of the intramedullary reaming were negative. He remains on lifelong oral fluconazole at 400 mg d^-1^; his *Coccidioides* CF titer remained unchanged at 1:4 . At last the follow-up, 4.5 years after the initial presentation, he was ambulating in a rocker-soled shoe with no signs of active infection.

## 4 Discussion

Coccidioidomycosis can produce a variety of musculoskeletal manifestations, as illustrated by these cases. While Cases 2 and 3 are consistent with the published literature demonstrating involvement of the knee and foot/ankle, the literature pertaining to coccidioidomycosis involving the hip joint is scarce (Ahmad et al., 2021; Li et al., 2014; Waterman et al., 2010; Weisenberg, 2018; Fishco and Blocher, 2000; Naeem et al., 2022; Nasrawi et al., 2020). While pulmonary coccidioidomycosis is common in the endemic area of the southwestern United States, the main risk factor for developing disseminated coccidioidomycosis is immunosuppression (Ampel, 2007; Stockamp and Thompson, 2016). This is consistent with Case 1 (HIV/AIDS) but Cases 2 and 3 demonstrate that apparently immunocompetent individuals can also develop musculoskeletal coccidioidomycosis, which has also been described previously (Rammaert et al., 2014; Reach et al., 2010). While diabetes mellitus has been associated with risk of severe pulmonary coccidioidomycosis, there is conflicting evidence on whether it is a risk factor for disseminated disease (Moni et al., 2023). In both Cases 2 and 3, it is possible that one or both had a non-infectious process that predisposed them to acquiring a coccidioidal infection because of the ongoing inflammation, a process described as locus minoris resistentiae.

These cases demonstrate the different manifestations of musculoskeletal coccidioidomycosis such as soft tissue abscesses, septic arthritis, and osteomyelitis complicated by osteonecrosis. Our second and third case demonstrate that a *Coccidioides* infection can mimic non-infectious pathologies such as osteoarthritis of the native joint or Charcot foot secondary to diabetes mellitus. Whether the third patient developed true Charcot that was subsequently infected or disseminated infection leading to Charcot-like bony destruction, the presentation could have easily led to premature closure without fungal workup. Missing the diagnosis of a *Coccidioides* infection can potentially lead to hardware failure and risk of limb loss, as seen in Case 3, or lead to progressive bone and soft tissue destruction, demonstrated in Cases 1 and 2. Case 2 was inappropriately diagnosed with osteoarthritis and received corticosteroid injections that worsened her fungal infection. Other studies have described *Coccidioides* infections being mistaken for inflammatory, degenerative, or neoplastic pathologies (Table 1) (Ahmad et al., 2021; Fishco and Blocher, 2000; Li et al., 2014; Nasrawi et al., 2020; Taxy and Kodros, 2005; Weisenberg, 2018).

Taking a thorough patient history, including travel history, to endemic areas can guide providers to screen for *Coccidioides*, but an increasing number of cases has been reported in non-endemic areas (Taxy and Kodros, 2005). If the prior history of pulmonary coccidioidomycosis in Case 3 had been noted earlier, then the diagnosis of his disseminated infection might have been recognized promptly. Among patients with septic arthritis, long-bone osteomyelitis, and tenosynovitis of the lower extremities in the *Coccidioides* endemic region, we recommend obtaining coccidioidal serology via EIA (enzyme immunoassay) and immunodiffusion techniques (which screens for IgM and IgG) with a reflex to the IgG complement fixation (CF) titers as part of the standard microbiologic workup, especially if the symptoms have been chronic (ranging from weeks to months), as opposed to bacterial infections of bones and joints that tend to present more acutely. Advanced medical imaging, including MRI and CT scans also play a significant role in establishing a diagnosis of coccidioidomycosis (Taljanovic and Adam, 2011). Coccidioidomycosis can be confirmed with tissue cultures and stains. Histopathology shows spherules and granulomas.

The *Coccidioides* CF test is a quantitative antibody assay traditionally used to monitor disease activity. As noted in Cases 1 and 3, CF titers remain unchanged despite surgical debridement. In a recent review, CF titers in disseminated disease may remain unchanged or stay elevated regardless of clinical improvement (McHardy et al., 2023). Some of these patients are serofast and others likely have a persistence of infection.

Musculoskeletal coccidioidomycosis often requires the surgical debridement of necrotic soft tissue and bone to reduce fungal burden and remove biofilm (Blair, 2007; Weisenberg, 2018; Ahmad et al., 2021). The existing literature describes multiple cases treated with the debridement of soft tissues, including muscle, synovium, fat, and meniscus (Ahmad et al., 2021; Li et al., 2014; Nasrawi et al., 2020; Taxy and Kodros, 2005; Weisenberg, 2018; Waterman et al., 2010). While the use of an amphotericin B spacer has been described in cases of *Coccidioides* prosthetic joint infection (PJI), there is a scarcity of data regarding use of antifungals mixed with polymethylmethacrylate (PMMA) or calcium sulfate beads. Cases 1 and 3 utilized calcium sulfate or methylmethacrylate beads impregnated with antifungals to fill significant dead space within the infected area. Typically, voriconazole (300–600 mg per batch of bone cement) or amphotericin B deoxycholate (recommend dose 1 mg kg^-1^ patient total body weight) is used. This allows a high concentration of antifungal drugs to address the infected tissues over a large surface area that may not have adequate vascular supply to deliver oral or intravenous antifungals. It is unclear whether beads with amphotericin B deoxycholate provide a clinical benefit over agents like voriconazole. In cases where there is significant osteonecrosis leading to joint collapse, as in Case 3, a PMMA spacer containing antifungal medications provided the benefit of additional joint rehabilitation. In addition to surgical management, patients with musculoskeletal coccidioidomycosis need close infectious disease follow-up, as prolonged antifungal therapy is often warranted. Triazole therapy is warranted for years, since active tissue destruction may recur when treatment is stopped (Galgiani and Kauffman, 2024). In a 1996 study (Dewsnup et al., 1996) of 18 patients for whom azole therapy for central nervous system coccidioidomycosis was stopped due to the presumption of a cure, 14 patients had a relapse with a disseminated disease. Extrapolating data from this study, coupled with our own institutional experience with musculoskeletal coccidioidomycosis, a relapse can have serious consequences, placing patients at risk for potential limb loss; thus, the recommendation is to continue indefinite triazole while utilizing a case-by-case approach to consider discontinuation following long-term anti-fungal suppression.

Even after extensive surgical and medical treatment, musculoskeletal coccidioidomycosis can still recrudesce in the same anatomical area, as demonstrated in Cases 1 and 3. It is worth noting that all three patients were treated with fluconazole at 400 mg daily at some point, which may be suboptimal for treating musculoskeletal coccidioidomycosis. A trial comparing fluconazole at 400 mg to itraconazole at 200 mg twice daily showed better results with itraconazole for treating skeletal coccidioidomycosis (Galgiani et al., 1993). We recommend checking the triazole drug level at least once after starting the therapy. The levels are not warranted if fluconazole is dosed at 800 mg d^-1^; however, we advise levels for voriconazole, itraconazole, posaconazole, and fluconazole at 400 mg d^-1^ in the setting of osteoarticular coccidioidomycosis.

## 5 Conclusions

Musculoskeletal coccidioidomycosis presents multiple challenges to treating physicians. Patients often present with symptoms suggestive of non-infectious etiologies, which can lead to misdiagnosis, inappropriate treatment, and devastating sequelae. While immunosuppression is a major risk factor, musculoskeletal coccidioidomycosis can occur in the immunocompetent. Treatment necessitates surgical debridement and long-term triazole regimens, yet recrudescence is not uncommon. Given the costly and debilitating consequences of missed diagnosis, physicians should have a low threshold for ordering fungal CF titers and fungal cultures from orthopedic specimens when managing patients with septic arthritis, osteomyelitis, and tenosynovitis of lower extremities in *Coccidioides* endemic regions.

## Data Availability

No code or data sets were used in this article.
